# Cyclodextrins and Cyclodextrin Derivatives as Green Char Promoters in Flame Retardants Formulations for Polymeric Materials. A Review

**DOI:** 10.3390/polym11040664

**Published:** 2019-04-11

**Authors:** Maria Paola Luda, Marco Zanetti

**Affiliations:** Dipartimento di Chimica, Università di Torino, Via P. Giuria 7, 10125 Torino, Italy; marco.zanetti@unito.it

**Keywords:** cyclodextrin, nanosponge, phosphorous fire retardant, char, inclusion complex

## Abstract

Polymers are intrinsically flammable materials; hence, fire retardance (FR) is required in their most common applications (i.e., electronic and construction, to mention some). Recently, it has been reported that cyclodextrin (CD) and cyclodextrin derivatives are beginning to be introduced into Intumescent Fire Retardant (IFR) formulations in place of pentaerythritol, which is used in IFRs that are currently on the market. Since IFRs are of less environmental concern than their hazardous halogen containing counterparts, the use of natural origin compounds in IFRs provides a way to comply with green chemistry issues. BCD and BCD derivatives presence in IFR mixtures promotes a higher yield of blowing gases and char when polymeric materials undergo combustion. Both processes play important roles in intumescence. The key rule to obtain in insulating compact char is the good dispersion of the nanoparticles in the matrix, which can be achieved by functionalizing nanoparticles with BCD derivatives. Moreover, CD derivatives are attractive because of their nanosized structure and their ability to form inclusion complexes with many compounds used as FR components, reducing their release to the environment during their shelf life of FR items. Often, fire retardance performed by BCD and BCD derivatives accompanies other relevant properties, such as improved mechanical resistance, washability resistance, self healing ability, thermal conductivity, etc. The application of CD fire retardant additives in many polymers, such as poly(lactic acid), poly(propylene), poly(vinyl acetate), poly(methyl methacrylate), linear low density poly(ethylene), polyamides, and polyesters are comprehensively reviewed here.

## 1. Introduction

### 1.1. Fire Safety

Intrinsically, polymers are highly flammable. Their extensive use in fields, such as construction, transportation, electrical and electronic, textiles, clothing, and so on, causes death or severe wounding of several people in fire events every year. From 2014 to 2016, an estimated 24,000 residential building electrical fires were reported to United States fire departments each year. These fires caused an estimated 310 deaths, 850 injuries, and $871 million in property loss [1]. Consequently, attention has been drawn to the fire-safety of polymers and an imposing body of normative information has been issued by the national and over national authorities in time. 

In Italy, DM 26/6/84 [2] defines criteria, regulation, and practice to classify and categorize materials in view of fire safety and prevention. Accordingly, the materials are divided into six classes; the higher the class, the greater the propensity to contrast the fire (non-combustible materials are in class 0). The UNI CEI EN ISO 13943/2017 similarly classifies materials according to their “fire reaction” in order to evaluate the role in fires of textiles, furniture, upholstery, wall and floor materials, and coatings. All countries in the world have increasingly strict requirements regarding fire safety, and the fire safety standards also have been continuously improved. In Europe, the EN 13501-1 [3] defines the fire classification procedure for all construction products, including those that were incorporated within building elements, such as floorings and linear pipe thermal insulation products. To meet fire safety standards, Underwriters’ Laboratories (UL) has written over 150 U.S. fire safety standards for flammability of plastic materials for parts in devices and appliances. The UL94 [4] provides flammability tests for polymeric materials as a preliminary indication of their acceptability with respect to fire safety for a particular application. In short, a flame of specified intensity was applied at the bottom of a sample of specified dimensions. Burning time and the dripping of flamed drops are considered in assigning the fire category (V-0, V-1, V-2, NC in decreasing order of fire safety performance, V-2 involves the dripping of flamed drops). Similarly, ASTM has issued hundreds of fire and flammability standards for testing and evaluating the ignition, burning, or combustion characteristics of materials that are used in interior and exterior building parts, household items, and furniture. In a candle test (limiting oxygen Index, LOI, ASTM D2863-97) [5], a sample is ignited from the top; LOI is the percentage of oxygen in a synthetic mixture O_2_/N_2_ that sustains combustion for at least three minutes. The cone calorimeter test reproduces, on the lab scale, the fire scenario simulating the development of a fire; it is widely used for testing the materials under development. To describe fire performance, the following main parameters are measured as a function of time according to ISO 5660 [6]: heat release rate (HRR, kW/m^2^), peak of heat release rate (PHRR), total heat release (THR, MJ/m^2^), mass loss (g/s), and smoke extinction area (SEA) smoke production rate (SPR, m^2^/s). The lower these parameters, the better the fire performance. Fire hazard is time related in principle, because it is not only the absolute values of combustion parameters that matter, but also whether they reach threatening values for damage to persons or property before or after evacuation time and/or the time required for fire brigade intervention. From this point of view, cone calorimetry is particularly useful in assessing fire hazard through time-related parameters, such as time to PHRR, but also indices, such as fire growth index (FIGRA, kW/s) and maximum averaged release rate of heat emission (MARHE, kW/m^2^) [7].

Fire and flammability standards, LOI, UL94, and cone calorimeter parameters are involved in the establishment of building codes, insurance requirements, and other fire regulations that manage the use of these materials and eliminate products with insufficient fire characteristics.

### 1.2. Fire Fighting Strategies

Developing an efficient, environmentally friendly, and universal fire-safe strategy for combustible polymers is a key goal, but it is quite challenging. At present, to improve the flame retardancy of polymers, the common method is incorporating flame retardants (FR) into the polymer matrices either as additive FR or chemically bonded to the polymer network (reactive FR). A Freedonia study [8] reported an annual increase of 5.4% in the global production volumes of flame retardants additives and estimated a production of a 2.6 Mton in 2016.

The complex process in which chemical and physical phenomena are interconnected across the condensed and gas phases during the combustion of a polymer constitute a Fire Loop with two connected chemical reactions mutually feeding each other: the condensed phase polymer pyrolysis and the gas phase oxidation of the issued volatiles. FRs can act in the gas phase by mainly quenching radical chain reactions, hence poisoning the flame. Some other FRs promote charring in condensed phase; some others again simultaneously exerted actions both in the gas and condensed phases [9]. A simplified kinetic model of the way to maximise this inherent cooperative effect, based on the respective effectiveness of the combined fire retardants, has been recently discussed [10]. The charring of the substrate has a twofold effect: (i) fixing within the residue part of carbon atoms of the polymer structure which otherwise would generate volatile fuel and heat in the combustion; (ii) protecting the surface of the burning polymer, as a shield that slows down the heat transfer from flame to the underlying polymer, and hamper fuel diffusion from the polymer to the flame. The effect of charring may be further enhanced if an expanded charred layer is formed; this is the so-called intumescence phenomenon. In an intumescent fire retardant formulation (IFR), a gas acting as blowing agent is simultaneously released with the charring process [11,12]. 

There are several categories of FR, based on their chemical structure and properties. Inorganic hydroxides (IH), halogenated flame retardants (HFR), and phosphorus-based flame retardants (PFR) are just three: IH are scarcely effective, need to be used in a huge amount with consequent detriment of mechanical performances; HFR efficiently poison the flame, but most of them have been phased out because of their toxicity. Unfortunately, the toxic potential of alternative halogen-free FRs is still rather unknown. Some PFRs, have been identified as replacements, acting either in the gas phase (poisoning effect of PO° radical) or in the condensed phase (promoting charring and intumescence of the substrate). Many of the PFRs used today are additive FRs rather than chemically bonded FRs; as a consequence, an easy release of FR from a polymer matrix to different environmental compartments via volatilization, leaching, and/or abrasion can occur [13,14]. However, information on the persistence, bioaccumulation, and toxicity of PFRs is limited. The toxicity of organophosphorous is strongly dependent on their structure and it varies widely. Some toxicity data can be found in literature about more common PFRs, such as triethylphosphate (TEP), triphenylphosphate (TPP), resorcinol bis(diphenyl phosphate (RDP), bisphenol A bis(diphenyl phosphate) (BADP), aluminum diethylphosphinate (ALPI), and 9,10-dihydro-9-oxa-10-phosphaphenanthrene-10-oxide (DOPO). Most of them in vitro reveal a low neurotoxicity [15,16,17,18,19,20]. On the other hand, some PFRs are considered to be carcinogenic and/or neurodevelopmental toxicants. There is increasing evidence that organophosphate triesters used as FRs can affect the thyroid system [21]. The decrease in concentration during the lifetime of additive FRs results in decreasing the fire safety of the FR protected items in time. An easy release to the environment of such compounds raises children’s exposure and that is of concern. Therefore, as a substitute of highly volatile PFRs, such as TEP or TPP, RDP, and BADP, are currently preferably used [22].


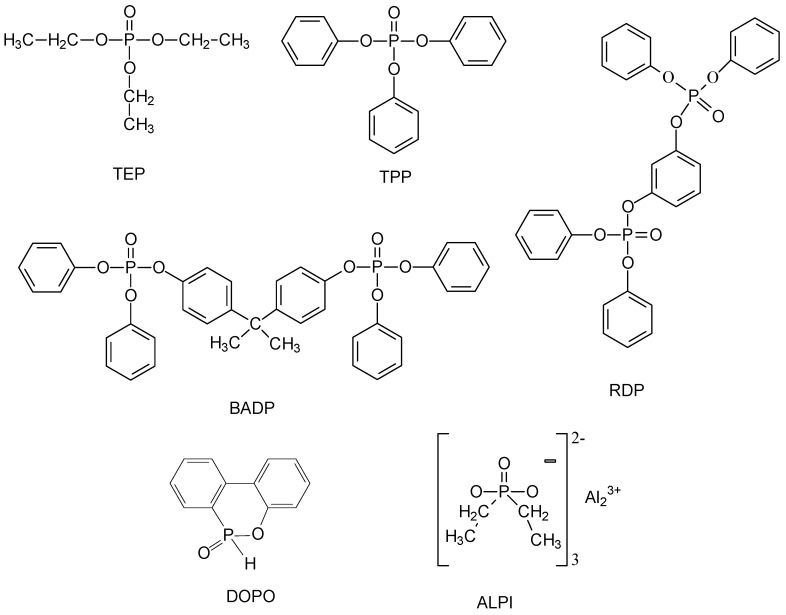


### 1.3. Environmental Concern and LCA of FR

Any manufacturer must comply with National and Overnational environmental legislation. Many standards for importation and commercialization of fire retarded items have been issued. For example, Directive 2015/863 [23], as published by the EU, (Restriction of Hazardous Substances, RoHS amendment 3) specifies that the maximum levels of 10 restricted substances must be 1000 ppm in electrical or electronic products, cables, components, or spare parts. Among these restricted substances, two halogenated FRs and four organic phthalates (Bis(2-Ethylhexyl) phthalate (DEHP), Benzyl butyl phthalate (BBP), Dibutyl phthalate (DBP), and Diisobutyl phthalate (DIBP) are included. 

In order to propose more environmentally friendly materials, the application of the Green Chemistry concepts leads in the last five years to the development of biobased additives for polymers [24]. One of the principles of Green Chemistry is the use of renewable raw matters: in fact, most of the commercially available FR components are oil-derived organic compounds, hence they have to face problems, such as the rising scarceness of oil, political and geological troubles, and impact on global warning. Therefore, in order to reduce the environmental footprint of FRs, renewable components in the intumescent compositions have been developed, mainly carbohydrates, such as chitosan, lignin, and starch [25,26,27]. 

Next to the intrinsic health and environmental properties of products, when synthesizing new biobased flame retardants, a life cycle assessment approach (LCA) should be used to evaluate the effective impact of these products. Biobased products have lower cradle-to-grave Greenhouse Gas Emission (GHG) and Non Renewable Energy Use (NREU) than the equivalent materials of fossil origin. Agricultural land use is obviously higher and it increases with biobased carbon content. Traditional oil based FRs production accounts for 5–45% of the cradle-to-grave GHG emissions of FR grade items. [28,29]. Therefore, the use of bio-based FR additives can effectively reduce the environmental footprint of the fire retarded products.

## 2. Cyclodextrin Role in FR Formulation

An improvement of the fire behavior for all the biobased flame retardant systems was essentially achieved through the charring effect. Among the renewable resources, lignin was probably the one providing the highest char yield at high temperature, and for this reason lignin was tested as single additive to improve the fire behavior of polymers [30]. However, lignin tends to reduce the time-to-ignition, increasing the ignitability of polymers, and this is penalizing in many classification tests. Alternatively, polyhydric compounds are good charring promoters in the presence of acid catalysts. Therefore, carbohydrates are considered to be worthy candidates in FR formulations and, among carbohydrates, chitosan is one of the more promising charring agents because of its high content of hydroxy and amino groups [31,32]. Tannic acid is a polyphenol that is very abundant in the vegetal kingdom and it exhibits excellent char-forming ability. Despite its large availability, tannins are not commonly used as FRs, possibly because they impart a dark color to their mixtures with polymers. 2% of tannin in epoxy foams decreases 20% of pHHR, however the mechanical properties are very much impaired [33]. Tartaric acid is another naturally abundant and non-toxic resource, its phosphinate and phosphonate esters displayed good flame retardancy in epoxy resins [34].

Phytic acid (Phy) is a natural polyphosphate esters of inositol that represents a common method of phosphorus storage in plant tissues. In mixture with chitosan, it imparted good fire retardance to ethylene-(vinyl acetate) copolymer (EVA) and polypropylene (PP) [33]. 


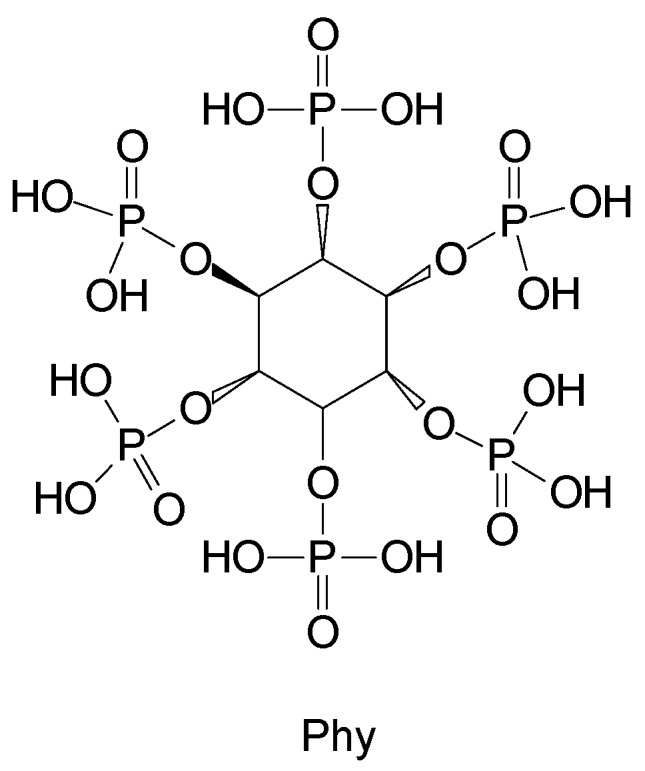


Several other natural resources have been tested as fire retardants in polymer, such as Deoxyribonucleic acid (DNA) and Ribonucleic acid (RNA) [33], isosorbide derivatives [35,36], gallic acid, and other multihydroxybenzoic acids [37], to mention a few. 

Cyclodextrins and cyclodextrin derivatives possess a huge number of hydroxyls and the charring process involves the opening of the cyclodextrin rings, followed by a chemical evolution similar to that of cellulose with a loss of the glucosidic structure and hydroxyl groups and buildup of unsaturation, carbonyl groups, and aromatic structures [38]. For this reason, they have also been tested as components in FR formulations [27].

Dextrins are the acid- or enzyme- catalyzed hydrolysis products of starch of different sources, such as potatoes, rice, and wheat; their formation depends on the type of bacteria digesting the starch. Cyclodextrins (CDs) are cyclic carbohydrate polymers that contain 6, 7, or 8 (1-4)-linked α-glucopyranose units in a toroidal shape ring, with a cone shape cavity. The larger and the smaller openings of the toroid expose to the solvent secondary and primary hydroxyl groups, respectively [39,40]. The treatment of starch with amylase from Bacillus Macerans gives a crude mixture of α- (∼60%), β- (∼20%) and γ-cyclodextrin (∼20%), together with small amounts of larger ring CDs and other impurities that are difficult to separate. The biotechnological advances from the ‘70s’ of last century dramatically improved their production: in 1970, β-cyclodextrin (BCD) was available at a price of about US$ 2000 per kg; the 2007 annual BCD production was close to 10,000 tons; and, the bulk price was lowered to about US$ 5 per kg, and it is expected to fall down more [41]. Of the three CDs, BCD is the widely used. Besides the high content of hydroxy groups that are available for charring, the most characteristic feature of CDs is their ability to form inclusion complexes with various molecules through host–guest interactions: BCD possess a lyophilic cavity with the right dimension (78 nm internal superior diameter) to host most of the organic molecules of pharmaceutical and food industry interests. These industries have exploited the encapsulation ability of BCD, but it is now under development in fire retardance, because a reduction of the release of volatile FRs to the environment in processing/using stages of FR products is expected. 


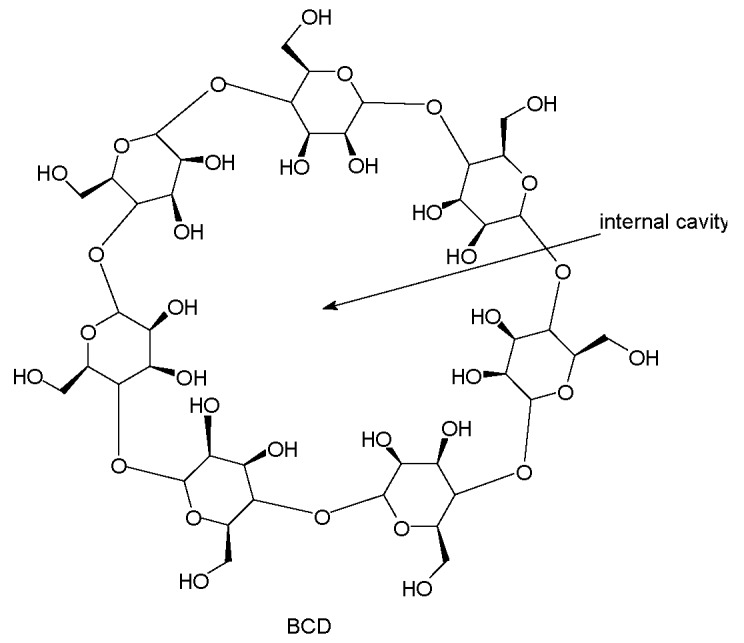


Native CDs are not able to incorporate large or certain hydrophilic molecules in their cavities, therefore several chemical modifications have been undertaken to overcome these limitations. Due to the six to eight reactive hydroxyl groups that are oriented towards the exterior side of the cavity, CDs act as monomers, cross-linkable with polyfunctional agents bearing hydroxy-matching groups, such as anhydrides, isocyanates, carbonates, epoxides, carboxylic acids, etc. The resulting insoluble three-dimensional covalent networks are the so-called CD-based nanosponges (CD-NSs) [42,43]. 


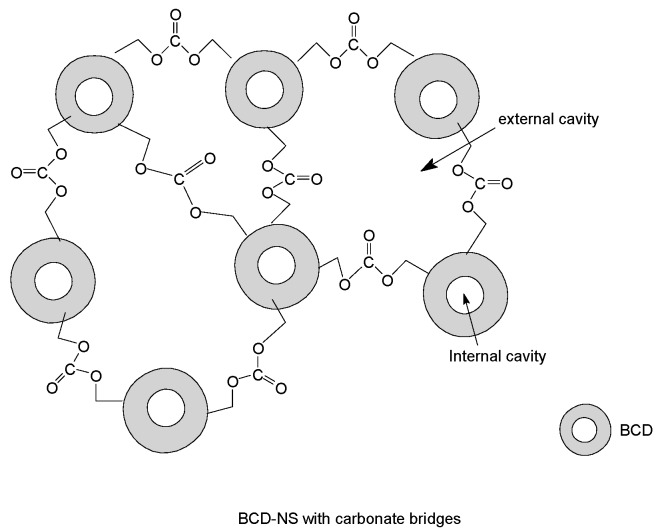


CD-NSs are highly porous nanoparticles; their final properties strongly depend on the nature of the cross-linker and degree of cross-linking [44,45]. CD-NSs incorporate molecules within their structure, either as inclusion complexes in the CD cavity or as non-inclusion complexes in the NS network with different and tunable polarities, thus enabling significant interactions between molecules that have different structures and lipophilicities. FR based either on BCD, modified BCD, or BCD-NSs prepared and tested in several polymers are described in the following.

### 2.1. BCD-and BCD Derivatives in Fire Retardant Formulation

**Figure 1:**
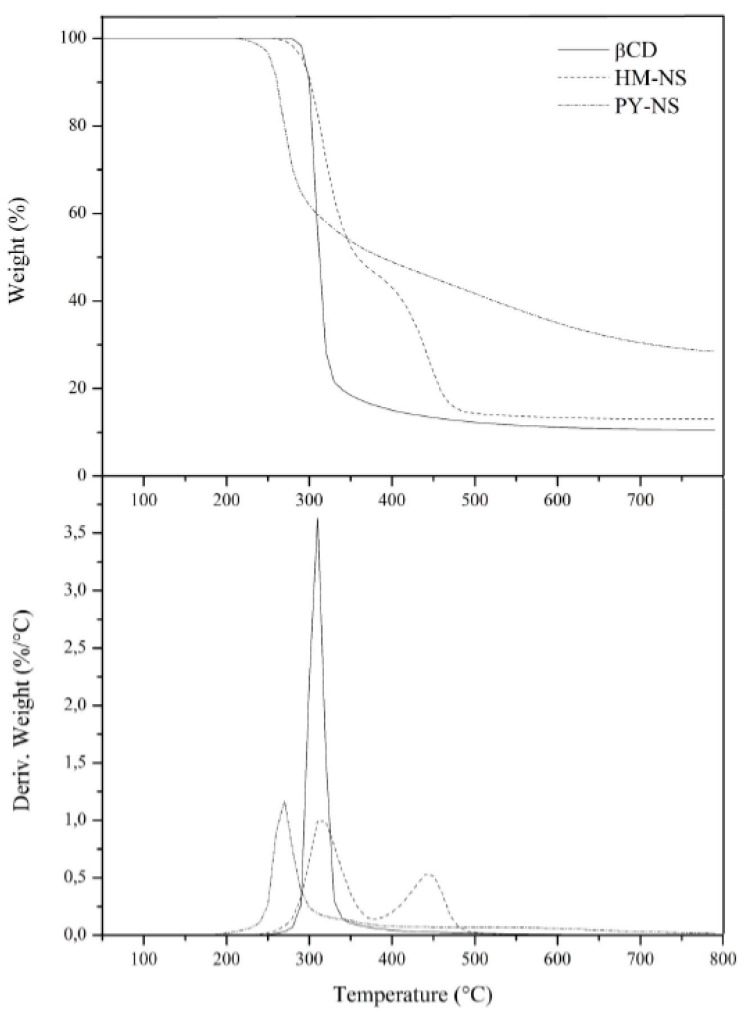
Thermogravimetry (TGA) (nitrogen flow, heating rate 10 °C/min) of β-cyclodextrin (BCD) and hexamethylenediisocyanate (HD-NS), pyromellitic dianhydride (PY-NS). Reprinted with permission from ref. [46].

Zanetti et al. [46] reported the thermogravimetric curves of BCD alone and of two BCD-NSs cross-linked, respectively, with pyromellitic dianhydride (PY-NS) or hexamethylenediisocyanate (HD-NS), as in Figure 1. BCD is not a great source of char, in fact, char yield was only at 9% at 800 °C. The amount of char raised to 10% and 28% for HD-NS and PY-NS, respectively, however PY-NS exhibited an early degradation in comparison to the two other compounds (220 °C instead of 270 °C). The char from PY-NS was shown to be highly porous, whereas that of HD-NS was not. 

Hence, in view of fire retardance, BCDs must be advantageously combined with phosphorus or nitrogen compounds to enhance charring. Otherwise, modified BCDs or BCD-NSs can replace simple BCD in FR formulations.

### 2.2. BCD and P or N Compounds

To our knowledge Le Bras et al. reported the first time BCD was used in FR formulation in 1997 [47]. FR performances in Linear Low Density Polyethylene (LDPE) of mixtures of ammonium pyrophosphate (PY) with xylitol (XOH) or d-sorbitol (SOH) or BCD were compared with the classical ammonium polyphosphate-pentaerythritol (APP/PER) intumescent based formulations. In this study, the additive ratio was kept to 30 wt.%, which allowed good fire retardant performances in the APP/PER system (LOI > 30%, UL94 rating V-0). In Table 1, the composition of the reported mixtures I-IV (Acid source/char forming column) refers to those having, for each formulation, the best fire retardant performances. It can be calculated that these compositions correspond to a P-OH/OH or PO^−^/OH molar ratio close to 1. In the case of BCD, this ratio is 1.1 when considering the primary –CH_2_OH only. Therefore, it appears that the best char formation involves reactions of these groups.


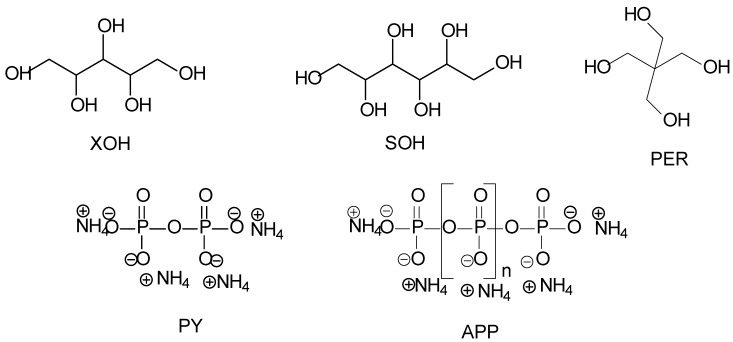


The results (Table 1) showed that, of the four mixtures, PY/BCD was that offering the worse FR performances in terms of LOI and UL94 tests (LOI 21%, UL 94 rating NC). This was attributed to: i) an early radical carbonization of BCD, already occurring during sample processing at 200 °C, inducing a premature radical degradation of LDPE. In effect, Raman spectra of its residue showed a higher presence of defect in comparison to that of the three other systems. ii) The contextual formation in BCD systems of a crystalline polyphosphate phase in the carbonaceous material, as shown by the XRD spectra, which makes it glassy and fragile. In contrast to the three others, in BCD systems, no phosphoric ester formation was observed, so preventing additional carbonization via phosphoric ester formation. 

Hence, BCD appeared not to be a carbon source of interest in intumescent FR additives for LDPE. However, the ability of BCD to generate a higher amount of char at high temperature than the SOH counterpart resulted in a cooperative effect in the PY/SOH/BCD system. The increased amount of “high temperature” residue was acting as a protective thermal barrier during the FR process, if the amount of BCD was relatively low to limit the mentioned drawbacks.

The charring contribute of BCD in fire retardance seems to be controversial, on the other hand Huang et al. first tested the effect in fire retardance of the inclusion capacity of BCD in Poly(ethylene terephthalate), PET [48]. The molecule that was selected to be hosted in BCD was a commercial FR Antiblaze RD-1, tri(methyl-di-ammoniumphosphonate) amine (FD) containing both P and N.


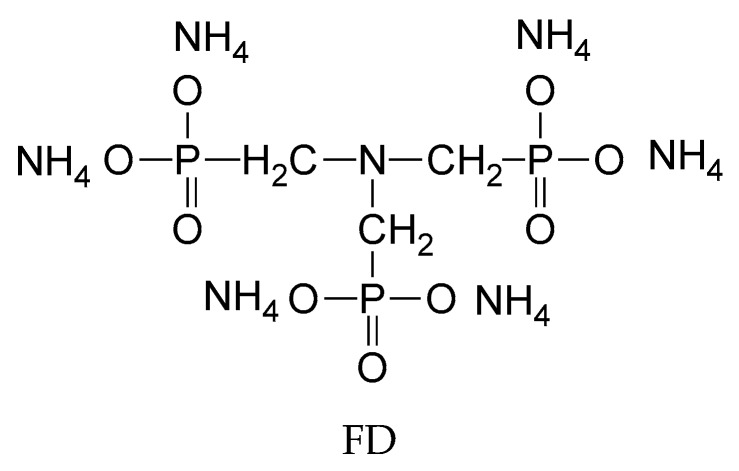


A cage herringbone inclusion complex, FD/BCD-CI, was successful prepared from the water solution and then melt-processed into PET (FD/BCD-CI-PET). Flammability was measured on films with a modified American Association of Textile Chemists and Colorists (AATCC) Test Method 34 [49] on PET containing the inclusion complex (FD/BCD-CI-PET) and, for comparison purposes, on simple PET, on PET melt mixed with 10% w BCD (BCD-PET), and on PET films soaked in FD solution and oven-cured at 180 °C for 5 min (FD-PET). The results (Table 2) demonstrated that all but the PET containing the inclusion complex were either completely or substantially consumed when ignited on a single edge with a standard flame for 3 s. 

In comparison to pure PET, BCD and FD decrease the burning time of the full length of the PET films, hence increasing their combustion rate. FD-PET behavior seems to depend on the amount of additive that is really caught during soaking. Opposite, FD/BCD-CI-PET soon extinguishes, greatly reducing its combustion rate. Thus, the efficacy of the inclusion complex in PET is not attributable to the presence of CD, but rather to the efficient disposition of the FR in the FD/BCD crystals that are embedded in PET.

Therefore, the BCD inclusion complex approach seems to be fruitful in the fire performance of polymers by fixing the involvement of the effective FR agent where and when really needed, thus eliminating unnecessary loss of FRs during the shelf life of FR goods. 

In most recent years, N. Zhang et al. [50] reached similar conclusions to Huang. He succeeds in the formation of an inclusion complex TPP: BCD 1:2 molar ratio, prepared by using a co-precipitation method from a BCD water solution and used it at a 10% load as FR for PET (BCD/TPP-CI-PET). To evaluate the outcomes of the inclusion complex, the flammability of films made with BCD/TPP-CI-PET was compared with those of a hot-pressed film of PET with 10% BCD (BCD-PET) or 10% TPP (TPP-PET) and with that of an untreated PET film. Flammability was measured according to a modified ASTM D6413 Standard test method for the flame resistance of textiles [51]. 

As shown in Table 3, the untreated PET and BCD-PET films burned entirely during the flame tests with intense dripping for both samples, with the rate of combustion of BCD-PET film being even faster than that of PET. Accordingly, BCD did not provide fire retardance properties. On the contrary, TPP-PET and BCD/TPP-CI-PET possess comparable flame resistant properties, becoming self-extinguishing soon after removing the ignition source. However, the effective weight of TPP in the PET-inclusion complex film was actually one-eighth of that in the TPP-PET film. As BCD did not provide fire resistance, the superior performance of the inclusion complex was attributed to the improved thermal stability of TPP encapsulated in the BCD cavities. The relatively small quantity of TPP that was present in the PET-CI film worked more efficiently than neat TPP, presumably inducing an extended shelf-life.

**Scheme 1:**
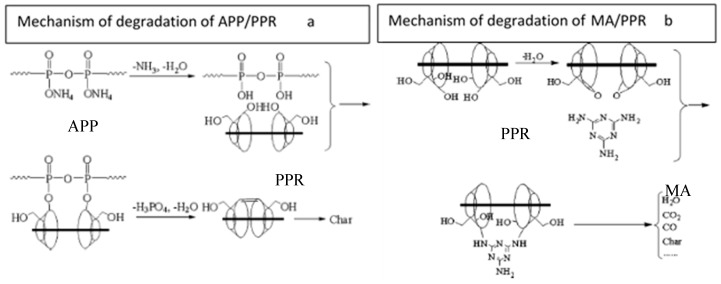
Mechanism of degradation of amine/ammoniumpolyphosphate–cross-poly(ethylenimine)/Polypseudorotaxane (APP/PPR) (**a**) and of melamine/PPR (MA/PPR) (**b**). Adapted with permission from ref. [52].

Wang et al. [52] prepared a Polypseudorotaxane (PPR) by the inclusion of BCD with polypropylenglycol (PPG) in water, and used it as a component in an IFR formulation to be introduced in poly(lactic acid) (PLA). SEM and Raman of chars that were obtained by melamine containing mixtures (MA/APP/PPR) and MA/APP/BCD proved that the char of inclusion complex PPR presented a better graphitic network than that of BCD. In PLA samples containing 15% of IFR, the LOI raised from 30 to 34.5% when PPR was used in place of simple BCD. The lower THR measured in cone calorimeter confirmed the better fire retardance behavior of samples containing PPR instead of BCD (from 43.1 to 48.0 MJ m^−2^, being 51.9 MJ m^−2^ that of pure PLA). A catalytic degradation of the PLA resin due to the acid species deriving from APP decomposition contributes to the formation of a charred layer. Additionally, the morphologies of the chars that were obtained in cone calorimeter tests of the two samples were different, that of MA/APP/BCD exhibited several holes and bubble, whilst that of mixtures containing PPR contained only few bubbles and so was providing a better flame shield for the material beneath. By combining previous literature data with their Raman, SEM, and thermal analysis results, the authors proposed a mechanism of degradation (Scheme 1): the BCD hydroxyl groups in the inclusion complex easily reacted with polyphosphoric acid (PPA) that formed from APP (Scheme 1a) and with MA (Scheme 1b), so promoting charring and foaming gases evolution. 

Apart for application in thermoplastics, BCD inclusion complexes were also introduced in thermosetting systems, where they were subjected to a curing process.

Very recently, Zhao et al. synthesized new P-N containing flame retardants molecules [53], N,N′-diamyl-p-phenylphosphonicdiamide (P-MA), and by Shan et al. [54], N,N’-dibutyl-phosphate diamide (DBPDA) to be used in FR formulations for epoxy resins (EP). 


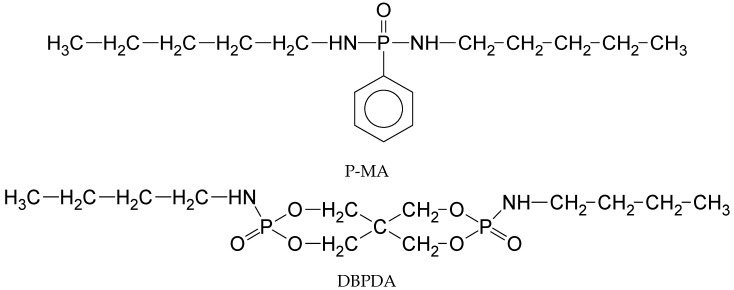


**Figure 2:**
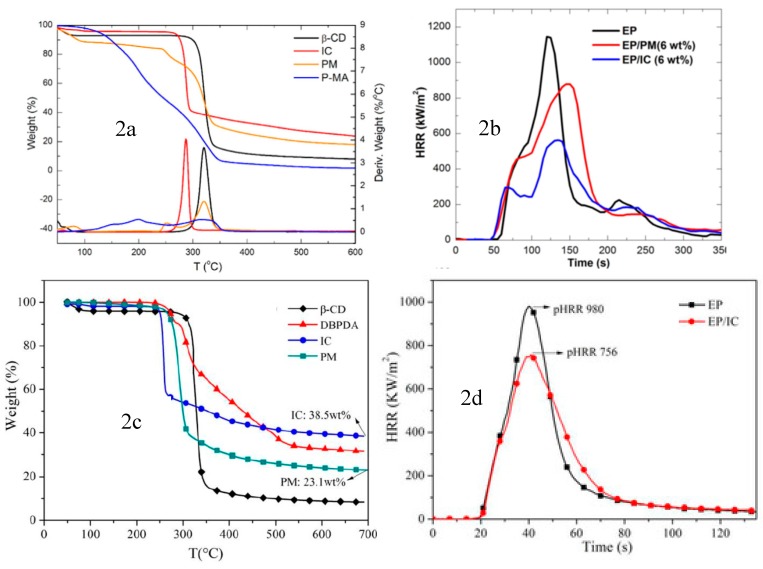
(**a**): TGA (Nitrogen flow, heating rate 10 °C/min) of N,N′-diamyl-p-phenylphosphonicdiamide (P-MA), BCD (β-CD), inclusion complex BCD-P-MA (IC); physical mixture BCD-P-MA (PM). (**b**): HRR from cone calorimeter test of epoxy resins (EP), EP fire retarded with 6% of BCD-P-MA physical mixture (EP/PM) and EP fire retarded with 6% of BCD-P-MA Inclusion complex (EP/IC). Adapted with permission form ref. [53]. (**c**): TGA (Nitrogen flow, heating rate 10 °C/min) of N,N’-dibutyl-phosphate diamide (DBPDA), BCD (β-CD), inclusion complex BCD-DBDPA (IC); physical mixture BCD-DBDPA (PM). (**d**): heat release rate (HRR) from cone calorimeter test of EP and of EP fire retarded with BCD-DBDPA Inclusion complex. Adapted from ref. [54] open access.

Each of them was assembled into the cavity of BCD, forming an inclusion complex. The physical mixtures BCD/P-MA and BCD/DBPDA were also prepared as well by co-dissolution in ethanol. Thermogravimetry (TGA) revealed that BCD assisted the thermal degradation of the P-N containing molecules, both in the inclusion complexes and in physical mixtures, inducing an earlier degradation and an increased amount of residue at 700 °C; however, this interaction was stronger in the inclusion complexes, eventually leaving even more residue, as in Figure 2a,c.

Further, 2–6 wt % of inclusion complex was dispersed into EP at 100 °C and then cured with the selected hardener at 160–200 °C (FR-EP). The fire retardance properties of FR-EP assessed by cone calorimetry tests revealed that the peak values of heat release rate (pHRR), Figure 2b,d, and smoke production rate (SPR) were reduced, suggesting that the inclusion complex could efficiently suppress the heat and smoke release. For instance, the reduction of pHRR was 23% by the addition of 3% of BCD/DBPDA inclusion complex, 24% by the addition of 6% BCD/P-MA physical mixture, and 51% by the addition of 6% BCD/P-MA inclusion complex. The use of inclusion complexes instead of MP was beneficial because of the stronger interaction of the components inside: BCD acts as a char source through catalytic action of phosphoric acid that was generated in situ from the P-MA or DBPDA component; meanwhile, the simultaneously released ammonia contributes to blow the residue. 

**Figure 3:**
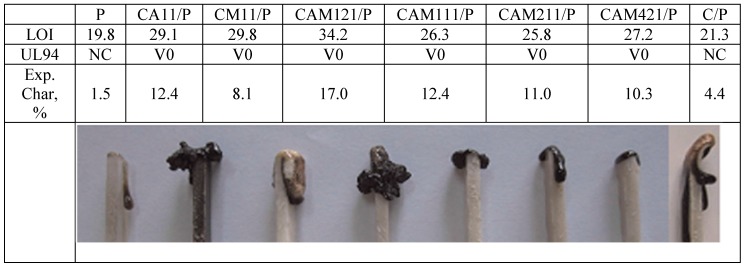
Photographs of specimens after limiting oxygen Index (LOI) tests of polylactic acid (PLA), 20% of fire retardant additive: CA11/P, CM11/P, CAM121/P, CAM111/P, CAM211/P, CAM421 /P, and CD/P.) and their LOI value and Underwriters’ Laboratories (UL) 94 rating. Symbol code: P: poly(lactic acid); C: cyclodextrin; A: ammonium Polyphosphate; M: Melamine. Number code: molar ration in the FR additive of C, A, M. Adapted with permission from ref. [55].

Apart for the positive effect on fire retardance, the preparation of inclusion complexes is often laborious, costly, and time consuming. Feng et al. [55] prepared mixtures by physically grinding BCD with APP and/or melamine (MA) to form a simple and greener FR complex replacing PER in the classical IFR formulation. 20 wt % additive was melt blended with PLA at 190 °C and hot-pressed to make specimens for UL-94 and LOI test. The results highlighted that BCD with an acid source, such as APP and/or blowing agent melamine MA, exhibited outstanding char-forming ability. Esterification occurring in APP/BCD systems or dehydration taking place in MA/BCD systems led to a higher char residue at 700 °C. Ternary systems APP/MA/BCD showed even larger char residue at 700 °C; the higher the amount of APP, the better the fire performances. The fire retardance of PLA was improved in the presence of a larger amount of char as it occurred in the presence of a relatively high content of APP. This char was able to stop further degradation in fire conditions (CAM 121/P, Figure 3).

One of the most simple and effective approaches to overcoming the PLA limitations, such as poor toughness, slow crystallization rate, and low thermal stability while enhancing the versatility of PLA bioplastics, was blending PLA with other miscible polymers, like polymethylmethacrylate (PMMA). However, as a depolymerizing polymer, PMMA exhibits poor fire performance due to its tendency to evolve quickly a massive amount of fuel, deteriorating the fire behavior of PLA in its blend. Teoh et al. [56] added 20% of a phosphate ester FR (isopropylated triaryl phosphate ester flame retardant, IPTAPE) to a PLA/PMMA blend (80/20 w/w) by simple melt compounding, attaining good UL94 rating (from V-2 to V-0) and LOI score (from 21.5 to 31.3%). Fire retardance was imputed to a condensed phase mechanism involving PMMA: the phosphoric acid that was released by IPTAPE was able to convert the ester groups of PMMA into acids and anhydrides, forming an effective barrier of carbonaceous char, which is able to increase polymer thermal stability and reduce rate and amount of volatile fuel. However, heterogeneous char morphology prompted the presence of a concurrent gas-phase mechanism in which most of the phosphorus was vaporized into gas phase during combustion. However, it was observed that the same self-extinguishing properties were achieved using as FR either 20% pure IPTAPE or a 20% mixture 1/1 w/w IPTAPE/BCD. Hence, BCD allowed a reduction of the global amount of phosphorous compound in the FR additive with no impairment of the fire retardant properties. Nevertheless, with pure IPTAPE, the mechanism was partially in the condensed phase with chemical involvement of phosphoric acid (formed from IPTAPE) and PMMA esters groups and partially in the gas phase by the evolution of phosphorylates species in the flame; on the other hand, with IPTAPE/BCD, the mechanism turned out to be only in the condensed phase. BCD, in the presence of IPTAPE, acted as a potential carbonizing agent for PLA/PMMA mixtures, because of its high amount of hydroxy groups that, by dehydration and decarboxylation reactions, assisted in char formation during burning without involving PMMA ester groups. As a result, a compact and wide coverage of char layer on the burning surface of PLA/PMMA/IPTAPE/BCD was formed.

Very good result on fire retardance of Poly(ether ester) elastomer (TPEE) was obtained by Zhang L. et al. [57], who used BCD, aluminum diethylphosphinate (ALPI), and melamine polyphosphate as fire retardant. BCD promoted the formation of stable and compact carbonaceous char, preventing the melt dripping. Moreover, the mechanical properties were quite good, at 25% wt FR loading. 

### 2.3. Functionalized BCD-in Fire Retardant Formulation

**Figure 4:**
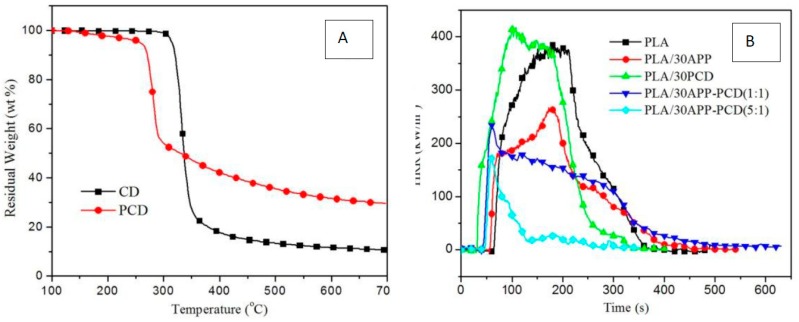
TGA of BCD (CD), and of phosphorylated BCD (PCD) (nitrogen flow, heating rate 20 °C/min); (**A**) Heat release rate in cone calorimeter test for PLA with 30% of various PCD/APP FR mixturesAPP/PCD in different ratio, (1:1 and 5:1); (**B**). Adapted with permission from ref. [58].

Many studies have been devoted to the chemical modification of bio-resources, with the aim of building new all-in-one biobased flame retardant molecules. A direct, in situ, generation of phosphoric acid and a better char-forming ability are expected if a phosphorous-containing agent is used to modify CDs. Among the possible chemical amendments, phosphorylation was probably the most frequently investigated. Zhang Y. et al. [58] modified BCD through interfacial condensation with a stoichiometric amount of phenyl phosphonic acid dichloride, obtaining a phosphorylated BCD (PCD). Even though the degree of the reaction of hydroxyl groups of BCD was not high, the charring ability of PCD was very much improved and a higher amount of char is produced. Figure 4A.

Notwithstanding the simultaneous presence of P and BCD in the same molecule and the higher amount of char, as seen in TGA, when PCD was melt mixed with PLA, the fire retardance was not improved (Figure 2B). The addition of APP in various ratio was highly beneficial to the fire retardance performance of PLA, as attested by LOI values, from 19.7% (neat PLA), to 30.4 (30% PCD/APP 1:1) or 42% (30% PCD/APP 5:1). Similar behavior is shown by UL94 rating, from NC (PLA and 30% PLA/PCD) to V-2 (30% APP) or V-0 (30% APP/PCD 1:1), and cone calorimeter evidences of Figure 4B. These results are not too different from those that were reported in ref. 55, where neat BCD and APP were used as FR in PLA at only 20% load (fig.3, BCD/APP 1:1, LOI 29.1%, UL 94 rating V-0,). However, with PCD, the surface of the intumescent char layer appeared more continuous and compact than that shown in Figure 3, and hence able to effectively hinder heat and fuel transport between polymer and flame. Carbonization involved preliminary reactions of the phosphoric acid from APP degradation with the residual hydroxy groups of PCD, which are responsible for the earlier degradation of PCD in TGA (Figure 4A) and for the greater amount of charred residue. APP plays the major role in fire retardance effectiveness: if the content of APP is low, then the protective char layer formed is not compact enough and does not hinder polymer combustion. 

### 2.4. BCD-NS in Fire Retardant Formulation

The encapsulation of APP and of other P containing products in a cross-linked BCD network is a strategy that is exploited in the fire retardance of many polymers. From one side, this way promotes charring, from the other, the active molecules are hosted in internal and/or external cavity of NS increasing their possible interaction. 

The microencapsulation of APP in cross-linked BCD network has been investigated in the fire retardance of PP/wood flour composites, WPC [59], and of ethylene-vinylacetate copolymer, EVA [60]. Fire retardance was improved in both cases and water leaching of the FR additive was reduced, thanks to its lower water solubility.

W. Wang et al. [59] cross-linked BCD with polydiphenylmethane diisocyanate (PMDI) to be used as a shell for microencapsulating APP (MCAPP), with the goal of improving the water durability of APP and preparing novel functional FRs via in situ polymerization. APP or MCAPP were mixed with wood-flour and PP to manufacture WPCs by hot pressing.

**Figure 5:**
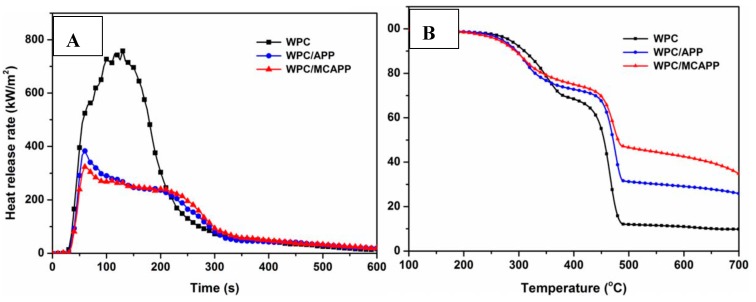
Cone calorimeter HRR (**A**) and TGA, nitrogen flow, heating rate 20 °C/min (**B**) of neat WPC, WPC/APP and miceroencapsulated sample WPC/microencapsulated ammonium polyphosphate (MCAPP). Adapted from ref. [59] with permission.

MCAPP provokes a higher residue (Figure 5B) and a better fire retardance behavior of the composite, as measured by HRR, UL94, and LOI, in comparison to neat WPC and WPC/APP (Figure 5A). The Raman spectra of the combustion residues of WPCs, through the ID/IG ratio, indicated that the microcrystalline size of the WPC/MCAPP residue was smaller than that of WPC/APP. The smaller the microcrystalline size, the greater the heat shielding efficiency. Thus, the comparatively rich charred residues with more compact microstructure rendered WPC/MCAPP more effectively flame retarded than the WPC/APP composite. Furthermore, the water solubility of APP after one hour was more than halved when incapsulated in MCAPP (from 0.358 to 0.172 g/100 ml at 25 °C and from 2.278 to 0.440 g/100 ml at 80 °C), demonstrating the barrier effect of the hydrophobic crosslinked BCD outside the microcapsule.

Similarly, B. Wang et al. [60] prepared BCD microencapsulated ammonium polyphosphate (MCAPP) by reaction between BCD, toluene-2,4-diisocyanate (TDI), and APP in one shot synthesis. The shell of the microsphere was made by cross-linked BCD-TDI, the core was APP and the core/shell ratio was 10–50%. Mixtures containing 35–40% of MCAPP in EVA were prepared in an internal mixer apparatus at 140 °C. The mixtures EVA/APP/BCD were also prepared for comparison purposes. Despite a relatively good LOI value (27–28%), these mixtures only attained V-2 rating in UL94 test as compared with those of their microencapsulated counterparts (UL94 rating V-0, LOI 28.5–32%). HRR measured in cone calorimeter confirmed these results: HRR from EVA/MCAPP was lower at any time than that of the EVA/APP/BCD blend, and they were both lower in comparison to neat EVA. Beside a better flame retardancy, microencapsulated samples also exhibited a higher thermal stability than EVA/APP/BCD, because CD shell improved the compatibility of the composites and the dispersion of APP in the EVA matrix. Furthermore, EVA/MCAPP composites demonstrate higher interfacial adhesion, mechanical, and dynamical mechanical properties than the EVA/APP/BCD composites.

In summary, the microencapsulation of APP with cyclodextrin may be a promising formulation for combining the acid source, the carbonization agent, and the blowing agent in one flame retardant, simultaneously satisfying the water resistance that is required in many industrial applications and solving compatibility problems.

With the aim of combining two strategies for flame retardancy, i.e., intumescence and use of nanoparticles, Alongi et al. [61] prepared a new flame retardant system containing a phosphorous compound and a stable nanosponge (NS). NS was synthesized by the crosslinking of BCD with an organic carbonate, thus forming a porous structure, including two types of cavities: the phosphorous moieties were entrapped either in internal cavities of BCD or in external cavities of NS, as shown by Raman and Thermal Analysis Studies. The resulting complex exhibited all features of an intumescent flame retardant system. Several phosphorous compounds were tested: TEP, TPP, APP, dibasic ammonium phosphate (APb), and diethyphosphoramidate (PhEt). 


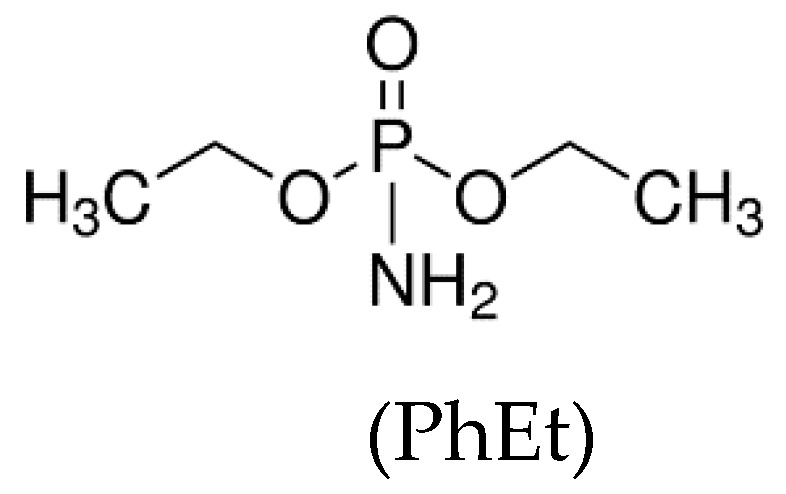


Phosphorous containing NS (NS-P) was incorporated in EVA and the fire properties were assessed. Regarding UL94, NS-P attained a V-2 rating in all cases; therefore, NS-P was not able to avoid dripping and long lasting combustion. Otherwise, in cone calorimeter test, 10 and 15 wt.% of NS-P enables a strong decrease of HRR, PHRR, and THR. Meanwhile, an anticipation of the ignition was registered (Table 4).

Alongi et al. also evaluated the FR effect of NS and of NS-P in PP, linear low density polyethylene, (LLDPE), and polyamide 6 (PA6) by mechanical grinding and compounding them with NS/P-NS in an internal mixer (at 190, 120, and 240 °C, respectively) [62]. NS/TEP 2:1 w/w or NS/APP 1:1 w/w (P-NS) were used as FR at 10 or 15% of total loading. The presence of both NS and P-NS did not affect the melting and crystallization temperature of the polymers, a presumable indication that there was no reduction of molecular weight during processing. On the contrary, as predictable, the crystallinity percentage was increased, because FRs acted as crystallization seeds during processing. 

As far as thermal stability is concerned, TGA analyses showed that, in the presence of NS and NS-P, PP and LLDPE degradation was starting earlier because of FR degradation, producing a foamed unstable char. The main LLDPE degradation remained substantially unchanged (482 °C), whereas that of PP was slightly postponed (from 463 to 476 °C). The amount of residue at 700 °C was larger, particularly when the P compound was APP (from >1% up to 3% in the case of LLDPE and from 0.5% up to 6% for PP). In contrast, in NS-P/PA6 systems, the main degradation step took place at a temperature that was very much lower (from 458 to 407 °C) because of interaction of the NS-P degradation products with PA6. 

Cone calorimetry attested an early ignition in all NS and NS-P systems, but only the formulations containing 15 wt.% of flame retardant NS-P were capable of strongly decreasing the HRR and PHRR. Decreasing the optical density of the fumes (SEA) was also observed. (Table 4). However, general improvement was a reduction of the burning rate (mm s^−1^) of fire retarder PP, LDPE, and PA6 in a vertical setup applying the flame at the bottom of the specimen (127 mm × 127 mm × 3.2 mm).

The evaluation of nonconventional additives as fire retardants on PA6,6, such as a master batch based on polyamide 6 and a halogen- and antimony-free flame retardant based on phosphorus, (CESA), α-zirconium dihydrogen phosphate, αZr(HPO_4_)_2_H2O, (ZrP), and BCD based nanosponges, NS, were investigated by Enescu et al. [63]. 

She carried out TGA studies in Nitrogen at heating rate of 10 C min^−1^ and, by comparing the experimental curves with the calculated ones (supposing no interaction between PA6,6 and additives), she observed that, in PA6,6/CESA composite, the polymer main degradation step was shifted to lower temperatures in comparison to that of neat polymer (temperature of maximum degradation 415 instead of 438 °C). On the contrary, it was shifted toward higher temperatures in PA6,6/ZrP and PA6,6/NS composites (452 °C in both samples). Meanwhile, an increase in the amount of the residue at 800 °C was also observed (12 and 5%, respectively, in comparison to 1 or 2% of PA6,6 and PA/CESA). To explain the thermal destabilization that occurs the in CESA composite, the reaction of the degradation products of PA6,6 with the degradation products of the additive was invoked. In contrast ZrP, acting as a solid acid, catalyzed the dehydrogenation of the polymer with the formation of carbon–carbon double bonds with a consequent stabilization of the polymer and formation of an insulating char.

Cone calorimetry data attested that heat release started in advance in all FR composites with a significant reduction of HRR for PA6,6/NS alone. This is usually indicative of a condensed phase mechanism of fire retardance. In composites fire retarded with traditional additives based on momtmorillonite MMT (PA6,6/C30B) and Carbon Nanotubes (PA6,6/MBCNT), however there was an even greater reduction of HRR with a significant enhancement of smoke production.

As seen, BCD is often used in FR formulation, however most of the polymers are rather hydrophobic, whereas the external surface of BCD is hydrophilic; their mixtures result is quite heterogeneous, and this is thought to impair the fire retardation effect in the whole system. Systems containing BCD-NS often display a better fire retardance behavior than is analogous with simple BCD.

Zhang et al. [64] prepared two BCD modified graphene (GO) nanomaterials: GO-CDs and GO-TDI-CDs. Fourier Transform Infrared Spectroscopy (FTIR), X-ray diffraction (XRD), TEM, SEM, and TGA characterized them. In a search for possible applications of these GO composites, their ability to dispersion in solvents and thermal stability were tested. They were dispersible in solvents, such ethylene glycol, because of similarity with GO and they were much more thermally stable than GO. Specifically, the introduction of CDs significantly raised the thermal stability of GO–CDs nanomaterials, because of strong inter- and intra-molecular interactions. In fact, about 30% of weight loss (onset temperature at 220 °C) was observed for GO, which was possibly due to the removal of water molecules absorbed and the oxygen-contained functional groups on GO, whereas there was nearly no weight loss before 220 °C for GO–CD. Hence, the introduction of CDs improves the thermal stability of nanomaterials and dispersion of the additive, confirming their potential in the FR industry.

To improve the mutual compatibility BCD/PP, Zheng et al. reacted 4, 4′-diphenylmethane diisocyanate (MDI), with BCD obtaining a crosslinked network (CM) [65]. SEM and molecular dynamics simulation confirmed the enhancement of compatibility between PP and CM. As far as PP fire retardance is concerned, beside CM, Melamine Phosphate (MP) and expandable graphite (EG) were considered as components in the FR formulations for PP. However, the flame retardancy of all binary mixtures was only slightly improved in comparison to that of neat PP (LOI 17.0%, UL94 rating NC with dripping of flamed drops) and none of them passed the UL-94 test. On the contrary, the PP/MP/CM system (30% FR additive MP/CM in various ratio) exhibited higher LOI and better UL94 rating (up to 28.3% and V-0 for MP/CM2/1). In comparison, PP with analogous MP/BCD 2/1 additive displayed lower LOI values (27.2%), indicating that the char-forming ability of BCD was enhanced via MDI modification. When 10% of the IFR mixture was replaced by EG, the fire behavior of PP composites burst, suddenly reaching 31.2%, LOI score, still keeping V-0 rating. FTIR of the residue at different temperature demonstrate that a cross-linking reaction between MP and CM occured with formation of a primary residue, which improves the onset of the PP degradation process. The esterification reaction between MP and CM accelerates the char-forming process at a low temperature. Simultaneously, the release of inert gases (NH_3_ and H_2_O from MP condensation and deammoniation) expanded char residue to some extent. Along with the swell effect of EG, a high-quality char residue system was further generated. This demonstrates the cooperative effect between IFR and EG in improving the thermal stability of the PP composites. 

**Figure 6:**
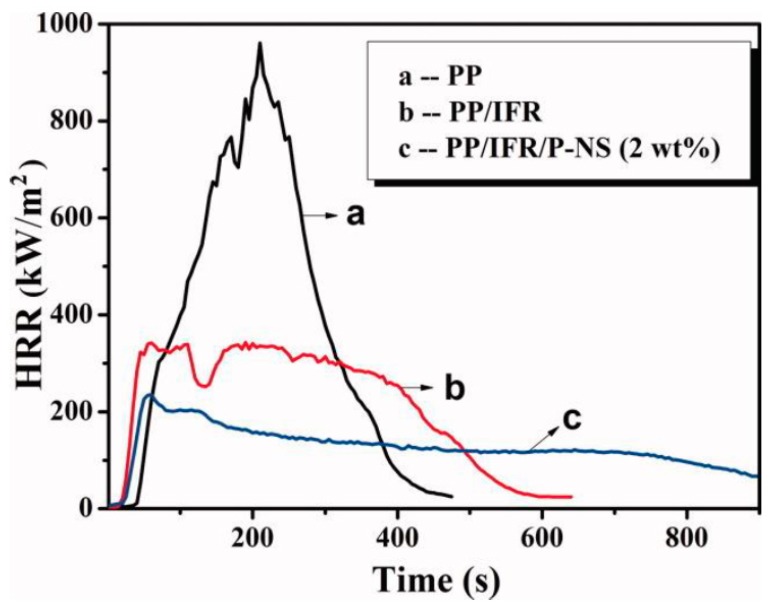
HRR of polypropylene (PP) (**a**), PP flame retarded with classical intumescent fire retardant formulation (IFR) formulation (**b**) and PP/IFR with 2% of P-NS as synergist. Reprinted with permission from ref. [66].

NS made by crosslinking BCD with an epoxy resins, (NS), as prepared by Lai et al. [66], was used for entrapping in its cavities resorcinol bis(diphenyl phosphate), RDP, a molecule with good potential for enhancing the flame retardancy, thanks to its high phosphorus content and good thermal stability. However, being very viscous, it was difficult to incorporate/retain in solid polymer composites. This phosphourous containing NS (NS-P) was compounded in a two rolls mixer with PP fire that was retarded with the classical intumescent formulation (Melamine pyrophosphate/PER, IFR/PP). NS-P showed an outstanding cooperative action in the fire retardant action of IFR/PP. For example, IFR/PP 25/75 exhibited LOI of 29% and UL94 rating V-1. With 2% of NS-P replacing IFR, the LOI increased to 32.5% and the UL94 rating was V-0. Cone calorimetry tests exhibited lower HRR with reduced ignition time (Figure 6).

**Figure 7:**
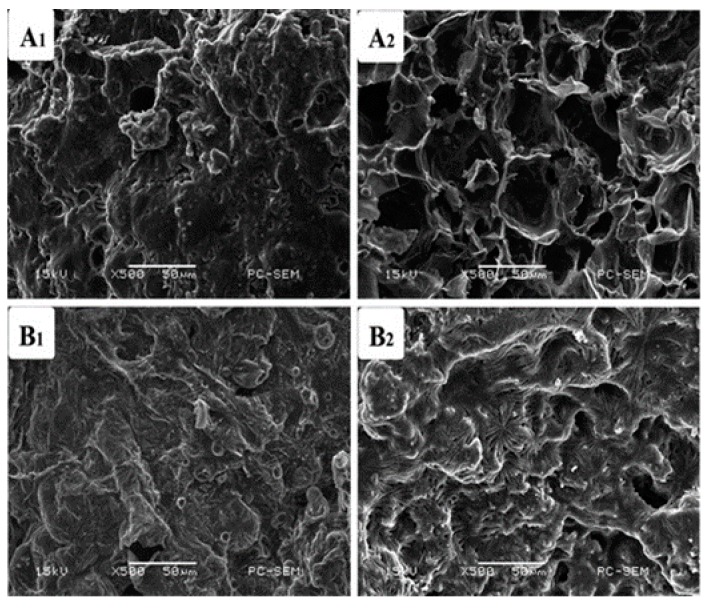
Scanning electron micrographs of the (**A_1_**,**B_1_**) upper surface and (**A_2_**,**B_2_**) the lower surface of the intumescent char obtained from the samples after cone calorimeter test: (**A**) did not contain P–NS in the PP/IFR and (**B**) contained 2 wt % P–NS in the PP/IFR. Reprinted with permission from ref. [66].

The intumescent char residue of the PP/IFR composite was rather fluffy, in contrast, incorporation of P– NS char was compact and hardly to be peeled off of the substrate. Finally, the morphology of the charred residue of FR PP revealed that the cooperative effect between P–NS and IFR on flame retardancy was due to the improvement in the formation of a compact and dense char barrier on the surface of the burning composites (Figure 7).

### 2.5. BCD System Grafted to Polymers

BCD is also attractive for use in fire retardance of textiles, because, beside its fire properties, it improves the functional properties and durability of the finished cotton fabrics. In this case, FR permanence and activity in the fabrics after several washing cycles is fundamental.

Kumar et al. and Veerappagounder et al. [67,68] prepared the monochlorotriazinyl-β-cyclodextrin (MBCD) by reacting BCD with cyanuric chloride. MBCD was then covalently grafted to cotton fabric with a high percentage of grafting (2.7%) (MBCD-gr-Cotton). Some cotton fabrics were also treated with 1,2,3,4-butane tetracarboxilic acid [60] or the cheaper, ecofriendly citric acid [67,69]. The flame retardant diammonium phosphate (DAP) was applied on MBCD-grafted cotton fabric and on the acid finished textiles. The resultant finished fabrics were compared for fire retardance functional properties and durability towards different washing cycles with untreated and differently treated samples. The flammability behavior and durability after 0, 1, 5, 10, 20, and 40 washing cycles was determined by LOI, and by measuring time and the length of burning according to ISO 6940-2004 [70]. The whiteness and loss of strength cyclodextrin grafted textiles were within the acceptable limits and the hand value alteration much reduced. Fire retardance was good in all DAP finished cotton fabrics, being LOI >31, and the burning rate was strongly reduced in comparison to the non-DAP treated samples. However, only DAP finished-acid treated-BCD grafted textiles retained their good fire performance upon washing, displaying an intumescent behavior resulting from the contemporary presence of charring agent (BCD), acid source (DAP), and foaming agent (Carboxylic acid). Durability was explained by the chemical bonding ability of carboxylic acid, either with DAP and BCD. 

### 2.6. Hybrid BCD Based FR

The combination of nanoparticles with conventional flame retardant additives, and especially with phosphorous-based ones, was reported to contribute to the enhancement of flame retardancy of polymers. Often, fire retardance in these systems goes together with other relevant properties, such as mechanical resistance, self-healing ability thermal conductivity, etc.

**Figure 8:**
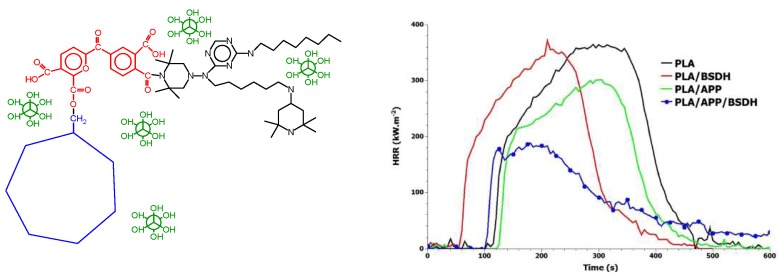
Structure of BSDH hybrid FR (**left**) and HRR for PLA and PLA with various FR additives (**right**). Reprinted with permission from ref. [71].

Recently, Vahabi et al. [71] developed a new halogen-free flame retardant by functionalizing BCD with a triazine ring containing polymer via an aromatic deanydrate that integrated with nanohydroxyapatite biomaterial into a hybrid FR system, BSDH (Figure 8, left). BSDH was applied as flame retardant for PLA; the fire performances of BSDH/PLA mixture were compared with those of APP/PLA and BSDH/APP/PLA mixtures. Nanoparticles were homogeneously dispersed within PLA matrix and cone calorimetric data highlighted that BSDH and APP showed a cooperative effect on improving the flame retardancy of PLA composites, greatly reducing HRR (Figure 8, right). This was attributed to the reduction of the amount of volatile compounds and to the formation of a thermally stable and very insulant residue. In the presence of PLA, APP played a catalytic role in char formation. However, the burning rate of PLA/APP/BSDH was very much lower than that of other systems thanks to the higher thermal stability, cohesion, morphology, thickness, and porosity of the char. Dehydrated nanohydroxyapatite contribute to the formation of this very insulating char.

Moreover, hydroxides based-layered double hydroxides (LDHs) are new attractive nanofillers. The chemical formula of LDHs can be [M^2+^_1−x_M^3+^_x_(OH)_2_]^x+^·[(A ^n−^)_x/n_·yH_2_O]^x−^, where M^2+^, M^3+^, and A^n−^ are divalent and trivalent metal cations and interlayer anions, respectively. They are suggested as flame-retardants additives, thanks to their endothermic decomposition at high temperatures. However, the improvement of polymer-based nanocomposites properties closely depends on the distribution of nanofiller in the polymer matrix. Biobased modifiers of the nanofiller with flame-retardant activity are preferably to this goal. Kalali et al. [72] selected Phytic acid, Phy, a natural resource compound that contains P, and a water-soluble cyclodextrin derivative (hydroxypropyl)sulfobutyl-β-cyclodextrin sodium (CDBS) as modifiers for LDHs. Furthermore, transition-metal oxides improved the thermal resistance of char residues and they served as catalysts for the cross-linking of macromolecules, increasing their final char yield. The incorporation of Fe_3_O_4_-CDBS-LDH hybrid into an epoxy matrix not only improved the flame-retardance and mechanical properties of EP, but also slightly increased the thermal conductivity (from 0.220 for EP and CDBS modified EP to 0.270 Wm^−1^·K^−1^ for Fe_3_O_4_-CDBS-LDH modified EP). These attractive performances were attributed to the unique structures, uniform dispersion states of the hybrids, and to the cooperative effect LDH/organic modifiers. 

Xuan et al. [73] prepared paper sheets that were coated with a multilayer films composed of poly(acrylic acid)-adamantamine (PAA-AD) and amine/ammoniumpolyphosphate–cross-poly(ethylenimine)-β-cyclodextrin (APP-co-PEI-BCD). BCD and AD are a host-guest pair having a high association constant. The substrate (paper or glass) was alternately immersed in aqueous solutions of PAA/AD and APP-co-PEI-BCD. Deposition was repeated via the host-guest interaction to obtain the double-network (PAA-AD/APPco-PEI-BCD). This coating made the paper self-healing without any initiating agents, thanks to the strong host (BCD) guest (adamantine fixed on PAA) interaction. Furthermore, this double layer system imparts flame retardant activity if sprayed on paper as coating. This is due to the contemporary presence of charring agent (BCD) and APP.

**Figure 9:**
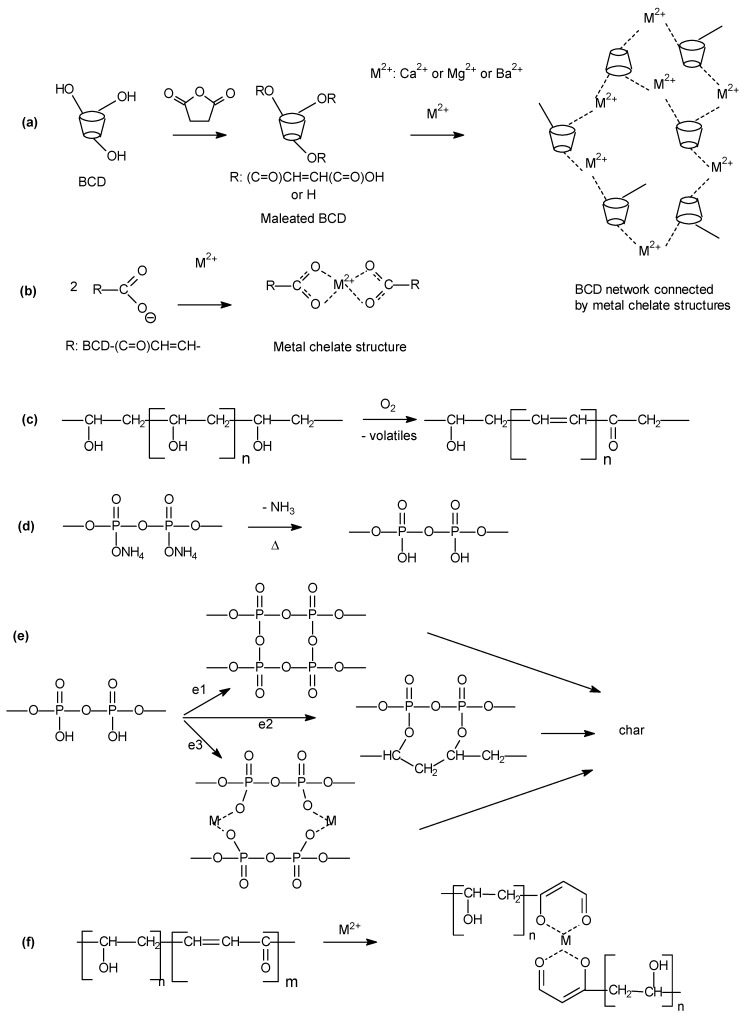
(**a**): Maleated BCD metal salt network; (**b**) Ionization of the Carboxylic Metal Complex. Possible reactions of char formation; (**c**): Poly(vinyl alcohol) (PVA) carbonization; (**d**) APP degradation to polyphosphoric acid (PPA); (**e1**) PPA self-crosslinking; (**e2**) PPA crosslinking with PVA; (**e3**) PPA crosslinking with metal MC complex; and, (**f**) formation of metal polymeric complex. Adapted with permission from ref. [74].

To increase the fire retardance of Poly(vinyl alcohol), PVA, a special modification of BCD was that investigated by Feng et al. [74]: In a PVA/APP mixture exhibiting intumescent behavior, PVA-IFR, with 15% wt total IFR amount, a maleated cyclodexdrin (MC), and its metal salts (Metal MC) were added in small amount (up to 3%) in the partial substitution of APP. The reaction between MC and metal forms a sort of BCD network that is connected by metal chelate structures with different bivalent metals (Mg, Ca, Ba), as in Figure 9a,b.

TGA analyses (carried out in air) showed that, in the presence of Metal-MC, the initial degradation of polyphosphoric acid (PPA) that is obtained from APP through reaction d in Figure 9 is postponed of 16 °C due to the additional stabilization effects of metal ions (reaction e3 in Figure 9, adding up reactions e1, e2. Furthermore, the metal MC enhanced the amount and the quality of the residue (which was more compact and not easy to crack). They proposed a reaction of char formation (reaction f in Figure 9), involving the conjugated carbonyl structure that is formed by the thermal oxidation-degradation of PVA (reaction c in Figure 9) and metal chelate; maleated BCD only acted as a support for metal ion complexes.

The UL-94 rating results indicated that a V-0 rating could be achieved when the total dosage of flame retardant was only 15 %wt. However, it was shown that 0.1–0.5 wt% metal MC in combination with 14.9–14.5 wt % APP was the suitable loading to obtain good flame retarded PVA composites. However, further investigation needs to support these hypotheses. 

BCD and Silicon containing compounds are attracting attention as new flame retardants. Hence, Wang et al. synthesized BCD containing silicone oligomers (CDS) [75], who compared flame retardancy of BCD/PP and CDS/PP composites.


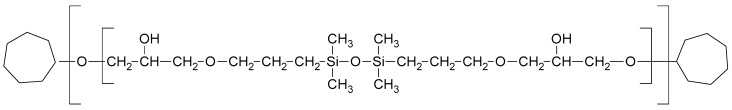


In order to obtain a better intumescent flame retardant system, CDS was also used as a synergistic agent in new intumescent flame retardant system containing a triazine polymer as char-forming agent (CFA) [76]. 25% loading of IFR consisting of CFA and APP 1:3 w/w showed very effective flame retardancy in PP, (LOI 32%, UL-94 rating V-0). However, in the presence of CDS partially replacing CFA in IFR (6.25 wt% of the IFR), LOI raised to 35.0% and UL-94 rating was still V-0, higher loading of CDS destroyed the char swelling behavior of the PP composites impairing flame retardance, indicating that there was an optimum synergistic concentration of CDS in the IFR systems. The char residue increased because of the interaction APP/CDS, since CDS, as thermally stable silicone, enhances the thermal stability of the charred residue. When compared with neat PP, the temperature of PP decomposition of the composites was higher, because of the char formation in IFR/PP. At the same time, CDS enhanced the mechanical properties of the composites, such as flexural strength and elongation at break, because of the toughness of epoxy silicon chain in CDS and of the better interfacial compatibility between IFR and PP. 

## 3. Perspectives

While some biobased FR components are actually on the market, to now all of the mentioned BCD fire retardance solutions are not scaled up yet, nor produced at an industrial level. Fire performance, easiness of processability, low toxicity, and preservation of other functional properties of polymers are just some of the main criteria that are to be taken in account in considering the convenience of scaling up these biobased FR.

Fire performance must be evaluated in respect of fulfillment of fire regulation specifications. In FR development, three main types of tests are usually carried out to characterize the flammability properties: LOI, UL94, and cone calorimetry, from which the main parameters can be extracted and introduced in the normative. As far as BCD based FRs are concerned, the key element is the achievement of the barrier effect through the promotion of a charred layer formation on the burning polymer, hence charring was generally related to lower pHRR and THR. Dripping of flamed droplets must also be avoided; however, UL94 V-2 rating, not a satisfactory rating for many applications, is often achieved with some BCD fire retarded polymers.

The protection of human health and environment must be promoted in any industrial business, which must comply with national and over national legislation. Several normatives restrict the use of some substances, the industrial development of new biobased FR must of course cope with these issues and with the REACH (Registration, Evaluation, Authorization, and Restriction of Chemicals) regulation. Moreover, natural products are not safe products by definition; their innocuousness must always be checked and a LCA approach should be used to determine the effective environment impact and the accomplishment of the green chemistry principles of new biobased FR. 

Due to the capacity of including some FR components of low molecular weight in their cavities, BCD and BCD derivatives reduce the release to the environment of these species, resulting in an increase fire retardance activity and in more environmental friendly behavior. Introduction in FR formulation of relatively high molecular weight maltodextrin endowed with inclusion capacity and relevant charring can also be investigated in view of their fire retardance application [77].

BCD based FRs that are introduced in a polymer matrix often produce a more homogeneous composite, showing better physical properties. Special morphologies, such as double layer films, microcapsules, or grafted systems are able to impose new properties to the resulting polymer composites, such as resistence to washability, thermal conductivity, and so on. Improvement in this field are highly welcomed. 

Eventually, economic criteria are at the basis of the industrial development of biobased FR. In this respect, low cost raw materials and low cost processes are pursued. Promising cheap raw materials are those that derive from well-established industries. BCD turn out to be more attractive, because of its wide employability in food, drug, and other industries, which make the cost of BCD nearly comparable to that of other biomolecules such as lignin or chitosan. Government policies can also affect industrial costs; for example, Sweden has recently introduced Tax on Certain Chemicals in Electronics (Electronics Law, 2016:1067), with 50%/90% tax reductions to apply to products that contain no halogenated compounds and phosphorus at load < 0.1 wt % This will enforce the industrial application of effective FR containing very low amount of P. On the other side, pollution charge, like a carbon tax, also supports virtuous environmental comportments. 

## 4. Conclusions

BCD were first used in flame retardants at the turn of the year 2000, but it was from 2010 when BCD was available on the market at a reasonable price, which studies on it very burst.

BCD alone is not suitable as a FR for any polymer. BCD and phosphorous-containing molecules (phosphate, phosphonates) promote fire retardance in polymers containing active functional groups, such as PET, PLA, Polyester elastomers, PMMA, epoxy resins, etc. by promoting the formation of a stable insulating char on the surface of the burning sample through interaction of the polymer and BCD with phosphoric acid that was developed from the phosphorous-containing molecules. Therefore, BCD, a natural origin resource, can replace molecules of fossil origin, such as PER in IFR formulation, hence meeting the green chemistry criteria. Where polymers are traditionally fire retarded with volatile phosphorous species acting in gas phase, the incapsulation of the phosphorous species into the cavity of BCD avoids the release of such compounds into the environment; at the same time, the amount of the residue increases and the load of phosphorous-containing species is reduced. However, fire retardance effectiveness is preserved or even increased and fire retardance turns out from a gas to a condensed-phase mechanism. Therefore, a more limited amount of P/N compounds are required to meet the fire retardance standard. However, due to the earlier radical charring of BCD, polyolefins, such as polyethylene, which contains little unsaturation (due to the synthesis), are not conveniently protected by the char that formed from the P/BDC FR system, at least according to the UL94 and LOI criteria. This is attributed to the occurrence of radical degradation reactions that reduce molecular weight and viscosity of the polymer promoting dripping of flamed droplets. 

Phosphorilated BCD do not improve fire retardacy very much, unless they are mixed with additional APP. This is due to the low degree of phosphorylation of BCD. On the contrary, fire retardance was enhanced in EVA or WPC composites by microencapsulating APP in a shell of crosslinked BCD. In this way, a better dispersion of APP was attained, as well as a microcrystalline size of the charred residue, which induces a better protection of the burning polymer substrate.

BCD-NSs effect on fire retardance of some polymeric matrices, such as polyamides, is able to react with phosphorous degradation moieties but has less effect on polyolefins. In this case, burning rate, THR, HRR, and SEA are diminished, so far reducing the fire risk. However, the UL94 rating is hardly affected, allowing, in most cases, the dripping of flamed polymer droplets. On the other hand, systems containing BCD-NSs often display a better fire retardance behavior than the analogous with simple BCD, because a better dispersion of the additive can be achieved in non-polar polymers. To improve the shielding capacity of the char, the addition of expandable graphite or graphene oxide or nanoparticles proved to be useful. Often, fire retardance accompanies other relevant properties, such as improved mechanical resistance, self healing ability, thermal conductivity, etc. The key rule to obtain an insulating compact char is the good dispersion of the nanoparticle in the matrix that can be achieved by functionalizing nanoparticles with BCD derivatives. 

## Figures and Tables

**Table 1:** Samples and fire retardance results for different char forming agent. Linear Low Density Polyethylene (LDPE) + 30% of fire retardant mixture (data adapted from ref. [47]).

Sample	Acid Source	Char Forming	Acid Source/Char Forming w/w	Catalyst	P-O-C (Ester Formation)	LOI %	UL94 Rating
I	APP	PER	3		YES	30	V-0
II	PY	XOH	2		YES	26	V-0
III	PY	SOH	2		YES	23.5	V-0
IV	PY	BCD	1		NO	21	NC
V	APP	PER	3	BCD °	n.d.	<30	n.d.
VI	PY	SOH	2.5	BCD *	n.d.	26	V-0

n.d.: not determined; ° any amount of BCD; * BCD 12.3%, PY 62% SOH 25.7%.

**Table 2:** Fire retardance performances according to a modified American Association of Textile Chemists and Colorists (AATCC) Test Method 34 for PET films and PET film with 10% of various FR additives. (data adapted from ref. [48], average of three tests).

Sample	Av. Film Thickness (mm)	Av. Char Length (cm)	Av. Burning Time after Remove of the Flame, s
PET untreated	0.14 ±0.01	BEL°	26.3 ± 1.5
BCD-PET	0.14±0.02	BEL °	16.3 ± 2.3
FD-PET ^a^	0.19±0.01	BEL° *	15.3 ± 1.2
FD/BCD-CI-PET	0.23±0.03	0.9 ± 0. 1	5.3 ± 1.2

^a^ 4–12% of FD; ° BEL= burned entire length, 10 cm; * one of the sample burned 5.3 cm.

**Table 3:** Fire retardance performances according to a Modified ASTM D6413: Standard test method for flame resistance of textiles. 10% load of various flame retardants (FR) additives. (data adapted from ref. [50], average of three tests).

Sample	Av. Film Thickness (mm)	Av. Char Length (cm)	Dripping	Av. Burning Time after Remove of the Flame, s
PET untreated	0.32 ± 0.01	BEL°	YES	25.7 ± 2.1
10% BCD-PET	0.39 ± 0.02	BEL °	YES	21.0 ± 1.7
10%TPP-PET	0.33 ± 0.01	1.9 ± 0.6	NO	3.3 ± 0.6
BCD/TPP-CI-PET	0.40 ± 0.01	2.59 ± 1.2	NO	3.3 ± 2.3

° BEL = burned entire length, 10 cm.

**Table 4:** Fire retardance performances of different polymers (data adapted from ref. [61,62]).

Polymer	%	NS	P	PHRR kW/m^2^	SEA m^2^/kg	TTI s	UL94 Rating	Burning Rate mm/min
EVA	100	-		1509		73	NC	
	95	5	-	1183		46	NC	
	95	3,3	1,7 TPP	1345		36	NC	
	95	3,3	1,7 TEP	1690		25	V-2	
	90	6,7	3,3 TEP	348		34	V-2	
	85		15 TEP	826		47	V-2	
	85	10	5 TEP	218		44	V-2	
	85		15 APP	981		46	NC	
	85	7,5	7,5 APP	239		39	V-2	
	85	7,5	7,5 APd	310		37	V-2	
	85	7,5	7,5 PhET	254		44	V-2	
PP	100	-	-	1541	609	46		1,8
	90	10		1462	551	34		2,0
	90	7	3 TEP	1529	457	30		1,4
	85	10	5 TEP	839	445	26		1,0
	85	7,5	7,5 APP	910	616	24		0,7
LLDPE	100			1823	450	64		0,9
	90	10		2063	364	34		0,6
	90	7	3TEP	2363	444	40		0,5
	85	10	5 TEP	1069	584	50		0,5
	85	7,5	7,5 APP	728	704	46		0,2
PA6	100			1111	134	78		2,2
	90	10		1196	128	28		1,1
	90	7	3 TEP	703	300	36		0,8
	85	10	5 TEP	1030	280	40		0,7

## Abbreviation

ALPI	aluminum diethylphosphinate
APb	dibasic ammonium phosphate
APP	ammonium polyphosphate
BADP	bisphenol A bis(diphenyl phosphate)
BCD	β-cyclodextrin
BCD-CI	β-cyclodextrin inclusion complex
BCD-NS	β-cyclodextrin bases nanosponge
BBP	Benzyl butyl phthalate
CD	cyclodextrin
CDS	silicone oligomer containing CD
CESA	phosphorous-based master batch
CFA	Char forming agent
CNT	Carbon Nanotubes
DAP	diammonium phosphate
DBP	Dibutyl phthalate
DBPDA	N,N’-dibutyl-phosphate diamide
DEHP	(Bis(2-Ethylhexyl) phthalate)
DIBP	Diisobutyl phthalate
DOPO	9,10-dihydro-9-oxa-10-phosphaphenanthrene-10-oxide
EG	expandable graphite
EP	epoxy resins
EVA	ethylene-vinylacetate copolymer
FD	tri(methyl-di-ammoniumphosphonate) amine
FD/BCD-CI-PET	β-cyclodextrin/tri(methyl-di-ammoniumphosphonate) amine inclusion complex in Poly(ethylene terephthalate)
FIGRA	fire growth index
FR	fire retardant, fire retardance
FR-EP	fire retardant, cured epoxy resin
GHG	Greenhouse Gas Emission
HD-NS	hexamethylenediisocyanate crosslinked β-cyclodextrin based nanosponge
HFR	halogenated flame retardants
HRR	heat release rate.
IH	Inorganic hydroxides
IFR	Intumescent Fire Retardant
IPTAPE	isopropylated triaryl phosphate ester flame retardant
LCA	life cycle assessment approach
LDPE	Linear Low Density Polyethylene
LLDPE	Linear Low Density Polyethylene
MA	melamine
MC	maleated cyclodexdrin
MCAPP	BCD microencapsulated ammonium polyphosphate
MARHE	maximum averaged release rate of heat emission
MDI	4, 4-diphenylmethane diisocyanate
MP	Melamine Phosphate
MTT	momtmorillonite
NREU	Non Renewable Energy Use
NS	nanosponge
NS-P	Phosphorous containing nanosponge
PA6	polyamide 6
PER	pentaerythritol
PET	Poly(ethylene terephthalate)
PLA	Poly(lactic acid)
PFR	phosphorus-based flame retardants
PhEt	diethylphosphoramidate
PHRR	peak of heat release rate
P-MA	p-phenylphosphonicdiamide
PMDI	polydiphenylmethane diisocyanate
PMMA	Polymethylmethacrylate
PP	Polypropylene
PPA	poly(phosphoric acid)
PPG	polypropylenglycol
PPR	Polypseudorotaxane
PVA	Poly(vinyl alcohol)
PY	ammonium pyrophosphate
PhY	Phytic acid
PY-NS	pyromellitic dianhydride crosslinked β-cyclodextrin based nanosponge
RDP	resorcinol bis(diphenyl phosphate)
SEA	Smoke Extinction Area
SPR	smoke production rate
SOH	sorbitol
TDI	toluene-2,4-diisocyanate
TEP	triethylphosphate
THR	total heat release
TPP	triphenylphosphate
TTI	time to ignition
UL	Underwriters Laboratories
V-0, V-1, V-2, NC	UL94 categories, in decreasing order of fire performance
WPC	polypropylene/wood flour composites
XOH	Xylitol
ZrP	α-zirconium dihydrogen phosphate
